# Exploring the Intestinal Microbiota Profile in Prostate Cancer Patients and Healthy Controls

**DOI:** 10.3390/microorganisms13092105

**Published:** 2025-09-09

**Authors:** Giovanna Cocomazzi, Annacandida Villani, Gandino Mencarelli, Viviana Contu, Daniele De Ruvo, Edy Virgili, Francesco Marino, Giorgio Maria Baldini, Elena Binda, Lodovico Parmegiani, Walter Ciampaglia, Lorenzo Capone, Francesco Perri, Antonio Cisternino, Valerio Pazienza, Concetta Panebianco

**Affiliations:** 1 Gastroenterology Unit, Fondazione IRCCS “Casa Sollievo della Sofferenza”, 71013 San Giovanni Rotondo, Italyv.pazienza@operapadrepio.it (V.P.); 2 Cancer Stem Cells Unit, Institute for Stem Cell Biology, Regenerative Medicine and Innovative Therapeutics (ISBReMIT), Fondazione IRCCS “Casa Sollievo della Sofferenza”, 71013 San Giovanni Rotondo, Italy; 3 Integrative Medicine Unit, Humanitas Gradenigo, 10153 Torino, Italy; 4 Gynaecology, Obstetrics and Reproductive Medicine Affidea Promea, 10126 Torino, Italy; 5 School of Biosciences and Veterinary Medicine, University of Camerino, 62032 Camerino, Italy; 6 Association for Research on Integrative Oncological Therapies (ARTOI), 00165 Roma, Italy; 7 Department of Biomedical Sciences and Human Oncology, University of Bari “Aldo Moro”, 70124 Bari, Italy; 8 Next Fertility GynePro, NextClinics International, 40124 Bologna, Italy; 9 Department of Urology, Fondazione IRCCS “Casa Sollievo della Sofferenza”, 71013 San Giovanni Rotondo, Italy

**Keywords:** prostate cancer, 16S rRNA gene sequencing, gut microbiota, bacterial signature, functional prediction

## Abstract

Recent studies suggest a role for the gut microbiota in the onset, progression, and prognosis of prostate cancer (PCa), one of the most common neoplasms in males. PCa screening relies on PSA testing, whose usefulness remains controversial due to its low specificity. This study was aimed at investigating the differences in the gut microbiota of PCa patients and healthy controls (HCs) and finding correlations between gut microbes and the clinical laboratory parameter assessed in the evaluation of PCa, to identify bacteria which could be used as diagnostic and prognostic biomarkers. Fecal samples collected from 18 PCa patients and 18 HCs were used to isolate bacterial DNA. 16S rRNA gene sequencing provided the gut microbial profiles of the enrolled subjects, whose functional impact was also predicted. A recursive partitioning tree method allowed us to identify a bacterial signature discriminating PCa from HC. A correlation analysis was performed between gut bacteria and the clinical laboratory parameters assessed in the evaluation of PCa. Differential bacterial patterns emerged between PCa patients and HCs, together with significant differences in beta-diversity, alpha-diversity, and richness. The functional prediction of the microbial profiles revealed several metabolic processes differentially regulated, including an enrichment in the Krebs cycle and in steroid hormone synthesis in PCa patients. A bacterial signature based on the abundance of *Lactobacillus* and *Collinsella* was found to discriminate between the two groups. Significant correlations were found between gut bacteria and the clinical laboratory parameters generally assessed in the evaluation of PCa. These results indicate that gut microbiota profiles may, in the future, represent potential biomarkers associated with prostate cancer risk or progression; however, further prospective studies and clinical validation are needed before considering their use as diagnostic or prognostic tools.

## 1. Introduction

Prostate cancer (PCa) is the second most common neoplasm in men and it is ranked as the fifth cause of tumor-related death in the world [1]. According to the Global Cancer Statistics 2022, the incidence of PCa varies by geographical area, being the highest in Northern Europe, Australia/New Zealand, the Caribbean, and Northern America and the lowest in Asia and Africa, reflecting the impact of genetics, lifestyle, and environmental elements as risk factors in the onset of the disease, but also the differences in diagnostic practices worldwide [1,2]. Beyond non-modifiable risk factors such as aging, ethnicity, genetic susceptibility, and family history, other potentially modifiable elements are proposed to favor PCa, including diet, obesity, cigarette smoking, alcohol consumption, chronic inflammation and prostatitis, and sex hormone levels [3]. The majority of these conditions are known to be intertwined with the composition of the gut microbiota [4,5,6,7,8,9,10], the trillions of microorganisms living in symbiosis with the human body and affecting host health and disease through different mechanisms. As observed for women, in which an unhealthy gut or vaginal flora are associated with cancer [11], recent studies suggest that imbalances in the microbiota have been linked to systemic inflammation, immune responses, and the metabolism of androgen hormones, all factors playing critical roles in prostate carcinogenesis [12]. For instance, an altered gut microbiota may increase the production of pro-inflammatory metabolites and carcinogenic compounds, potentially creating an environment conducive to tumor development [13]. Other studies in mice and in humans have suggested the ability of the gut microbiota to influence PCa progression by affecting testosterone levels and other androgens, hormones closely associated with prostate cell growth [9,10,14].

PCa screening can pose difficulties due to the limits of prostate-specific antigen (PSA) testing which, though widely spread and useful in early detection, can produce a considerable number of false positives, being also elevated in non-cancerous conditions, including prostatitis, benign prostatic hyperplasia, and urinary tract infections [15]. The diagnostic confirmation of PCa is based on the histologic evaluation of a prostate needle biopsy [16], which also allows us to establish a prognosis through the assignation of a Gleason score representing the tumor grade [17].

For the above-mentioned reasons, investigating the bidirectional relationship between the gut microbiota and PCa may pave the way to the development of new screening, diagnostic, prognostic, and therapeutic tools, including the use of probiotics, prebiotics, and fecal microbiota transplantations to modulate the gut microbial composition and potentially impact the disease course.

In this work, we aimed to assess whether the gut microbial profile of PCa patients could be discriminating as compared to the one of HCs, investigating also the putative functional mechanisms affected and the microbial association with the main clinical parameters assessed in the screening, diagnosis, prognosis, and follow-up of PCa patients.

## 2. Material and Methods

### 2.1. Study Population

PCa patients and HCs were recruited at Fondazione IRCCS “Casa Sollievo della Sofferenza” Hospital, San Giovanni Rotondo, Italy, after receiving ethical approval from the Ethical Committee (approval number N.184/CE, 15 March 2019). Written informed consent was obtained from the study participants and the same protocols for each patient regarding the collection, processing, and conservation of biological samples were followed. The study was carried out in accordance with the Declaration of Helsinki and in compliance with national legislation following the principles of good clinical practice. Anthropometric, lifestyle, and clinical–pathological data were collected at the time of recruitment together with the stool specimen collection. Only patients with PCa identified at the time of diagnosis prior to any cancer treatment were included in the study. Both PCa patients and healthy controls had not been taking antibiotics for at least 3 months prior to enrolment.

### 2.2. Laboratory Test Analysis

At enrollment, PCa patients underwent clinical laboratory blood tests, including blood cell count, serum protein and albumin levels, liver and kidney function tests, coagulation tests, inflammatory markers, and prostate-specific markers. These tests were performed according to clinical routine at accredited laboratories at the Fondazione IRCCS “Casa Sollievo della Sofferenza” Hospital, San Giovanni Rotondo, Italy.

### 2.3. Bacterial DNA Extraction from Fecal Samples

At enrollment, each study participant collected a fecal sample in a tube filled with a DNA stabilization buffer (Stool Sample Collection & Stabilization Kit, Canvax, Valladolid, Spain). Bacterial DNA was isolated using the QIAamp Fast DNA Stool Mini Kit (Qiagen, Hilden, Germany) according to the manufacturer’s instructions. DNA was quantified through a NanoDrop spectrophotometer and then stored at −20 °C until further use.

### 2.4. 16S rRNA Gene Sequencing

High-throughput sequencing of the V3–V4 hypervariable region of bacterial 16S rRNA gene was performed according to the Illumina 16S Metagenomic Sequencing Library Preparation instructions, as detailed in [18]. Briefly, amplicons were generated through a PCR step with universal primers, univocally barcoded using a dual-index system in a second PCR step, and pooled at equimolar concentrations. Paired-end sequencing of the pooled library was performed on an Illumina MiSeq device (2 × 300 cycles), Illumina, Hayward, CA, USA. Sequences were de-multiplexed based on index sequences, and FASTQ files were generated for each sample. FASTQ files containing 16S rRNA gene sequencing raw data were deposited in the repository ArrayExpress under the accession code E-MTAB-14719.

### 2.5. Bioinformatic Analysis

Gut microbiota raw data (FASTQ) were processed in QIIME2 v.2024.2 [19,20]. Illumina primers (forward and reverse) were trimmed with q2-cutadapt [21], q2-dada2 [22] was applied, and ASVs (amplicon sequence variants) were obtained. Taxonomy was assigned against the ASVs with the q2-feature-classifier plugin [23] using SILVA 138.1 99% OTUs (operational taxonomic units) Naïve Bayes classifier [24,25]. Alpha diversity indices (Shannon, Simpson, Chao1) were calculated with q2-diversity, and visualized and tested using R (version 4.3.2). Beta diversity was calculated using Bray–Curtis dissimilarity and visualized with PCoA. Differential abundance of taxonomic assignments and KEGG pathways, obtained using Tax4Fun2 [26] in R, was tested between groups. Taxa and pathways with a relative abundance <0.001 in at least one group mean were excluded. Pathway results were graphed using STAMP v2.1.3 [27]. The bacterial genera differentially represented between the two groups were analyzed using a recursive partitioning tree [28] to assess classification performance with absolute values, previously added with a pseudocount of 1, and natural logarithmic transformation. The analysis was performed using “rpart” package in R environment. Spearman’s correlation analyses between clinical parameters and gut bacteria were performed using GraphPad Prism 10.4.2 and visualized as correlation matrix.

### 2.6. Statistical Analysis

The anthropometric, lifestyle, and clinical characteristics of the subjects enrolled in the study were reported as means and standard deviations, medians and interquartile ranges, and frequencies (both absolute and percentages) for continuous and categorical variables, respectively. Comparisons between the two groups were performed using a two-sample *t*-test, Mann–Whitney U test, or the Chi-Square test with Yates’ continuity correction, as appropriate. Concerning the microbiota analysis, the alpha-diversity indices were tested with Mann–Whitney test, considering *p* < 0.05 as significant. Bray–Curtis beta-diversity was tested for group differences using PERMANOVA, with *p* < 0.05 being significant.

The performance of the bacterial signature was analyzed, calculating ROC curves, sensitivity, specificity, and confidence intervals. These analyses were carried out in R (version 4.3.2), employing the rpart, pROC, and caret packages. As for the prediction of bacterial functional pathways, significance was determined by a Mann–Whitney test, and P-values were corrected using the Benjamini–Hochberg method (corrected *p* < 0.05). Spearman’s correlations between clinical parameters and gut bacteria were performed using GraphPad Prism 10.4.2, visualized as correlation matrices and considered significant when *p* < 0.05.

## 3. Results

### 3.1. Study Population

Eighteen patients suffering from PCa and eighteen HCs were enrolled in the study. The anthropometric and lifestyle data are reported in Table 1. The two groups were balanced in terms of height, body weight, BMI, diet, attitude towards smoking, and alcohol consumption. A little, though significant, difference concerned age, with PCa patients being older than controls, despite both groups of subjects being, on average, in their sixties.

### 3.2. Comparison of Gut Microbiota Composition of PCa Patients Versus HCs

**Figure 1:**
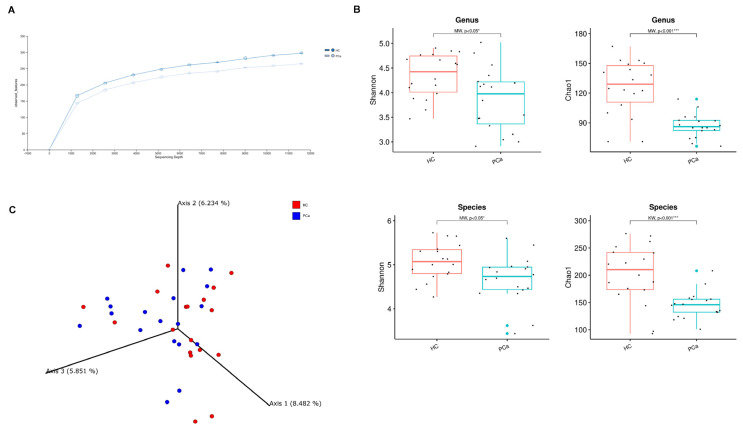
Gut microbial diversity in PCa and HC. Rarefaction curves for the analysis of microbial species content in the two groups of subjects (**A**). Microbial community a-diversity indices (Shannon and Chao1) of the two groups at genus (**top**) and species level (**bottom**). *p*-values from Mann–Whitney test are shown. * indicates *p*-value < 0.05; *** indicates *p*-value < 0.001 (**B**). PCoA showing beta diversity based on Bray–Curtis dissimilarity (**C**).

To explore the differences in the gut microbiota of PCa patients and HCs, 16S rRNA gene sequencing was performed and the results were compared between the two groups. The rarefaction curves in Figure 1A show a suitable amount of sequencing data for obtaining a comprehensive analysis in both the experimental groups. The gut bacterial richness, expressed by the Chao1 index, was found to be significantly lower in PCa patients compared to HCs starting both at the genus and species levels. Similarly, the alpha-diversity metric Shannon index was significantly lower in PCa versus HCs both at the genus and species level (Figure 1B).

**Figure 2:**
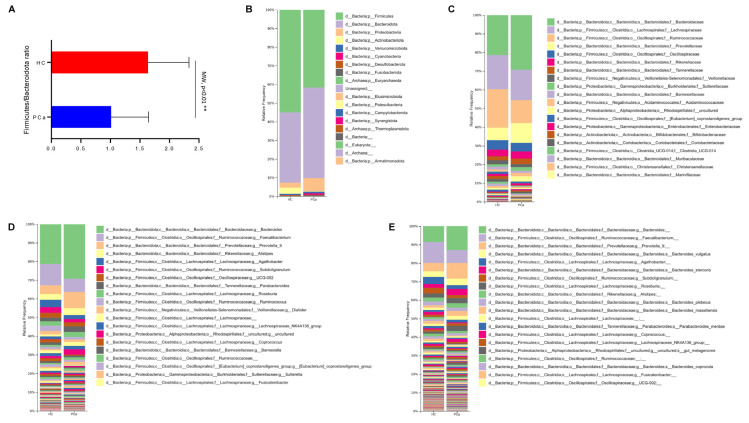
Gut microbiota composition in PCa and HC. Ratio between Firmicutes and Bacteroidetes in the two groups. *p*-value from Mann–Whitney test is shown. ** indicates *p*-value < 0.01 (**A**). Barplots representing the relative abundance of gut microbes at the phylum (**B**), family (**C**), genus (**D**), and species (**E**) level in the two groups. On each legend, only the top 20 bacteria were reported.

Concerning the beta-diversity, the analysis of the Bray–Curtis dissimilarity matrix revealed a significant clustering between PCa and HCs (Figure 1C). The compositional analysis of the gut microbiota in the two groups of subjects also showed significant differences. First of all, the Firmicutes/Bacteroidetes ratio, which is a marker of dysbiosis, was found to be decreased in patients compared to controls (Figure 2A). Indeed, both phyla were significantly changed between the two groups, with Firmicutes decreased (42.1% vs. 56.4%) and Bacteroidetes increased (49.3% vs. 37.9%) in PCa as compared to HCs; in addition, the phylum Actinobacteria was also under-represented (0.27% vs. 1.94%) in PCa in comparison to HCs (Figure 2B). A significant drop was observed in PCa for the families Ruminococcaceae (12.8% vs. 21.0%), Coriobacteriaceae (0.03% vs. 0.80%), Eggerthellaceae (0.01% vs. 0.11%), Erysipelatoclostridiaceae (0.05% vs. 1.17%), and Muribaculaceae (0.15% vs. 1.06%), whereas a rise was found for Lactobacillaceae (0.87% vs. 0.02%) with respect to HCs (Figure 2C). At the genus level (Figure 2D), *Collinsella* (0.03% vs. 0.80%), Erysipelotrichaceae_UCG-003 (0.03% vs. 0.16%), *Coprococcus* (0.59% vs. 1.64%), *Butyricimonas* (0.09% vs. 0.32%), and *Fusicatenibacter* (0.44% vs. 1.06%) significantly decreased, while *Lactobacillus* (0.81% vs. 0.01%), *Prevotella* 9 (10.0% vs. 3.8%), and *Anaerostipes* (0.12% vs. 0.03%) increased in PCa compared to HCs. Finally, among the species (Figure 2E), *Bacteroides eggerthii* was found to be significantly under-represented (0.392% vs. 0.567%), whereas Lactobacillus jensenii (0.157% vs. 0.002%), *Alistipes finegoldii* (0.292% vs. 0.038%), and *Bacteroides fragilis* (0.328% vs. 0.099%) were over-represented in PCa with respect to HCs.

### 3.3. Predicting the Functional Capabilities of Microbial Communities Based on 16S Datasets

**Figure 3:**
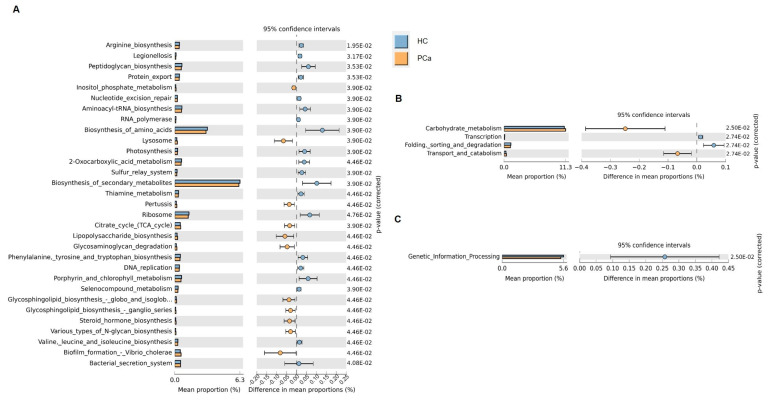
Prediction of gut microbial functions. Significant relative abundance of predicted function (mean proportions %) and different abundance of predicted function (difference in mean proportion %) for KEGG modules level 1 (**A**), level 2 (**B**), and level 3 (**C**) in pairwise comparisons between PCa and HC. FDR-adjusted *p*-values from Mann–Whitney test are shown.

Based on the 16S rRNA gene sequences, the functional profile of the bacterial communities in the gut of the two cohorts of subjects was predicted using Tax4Fun2_1.1.5 software. Several functional pathways were differentially represented between PCa and HCs, according to the level 1 KEGG module (Figure 3A). The biosynthesis of many amino acids was found enriched in HCs, whereas the Krebs cycle, and the biosynthesis of steroid hormones and of glycosphingolipids were higher in PCa. In addition, the carbohydrate metabolism and transport and catabolism were enriched, whereas transcription and folding, sorting, and degradation were under-represented in PCa patients according to the level 2 KEGG module (Figure 3B). Finally, genetic information processing was enriched in HCs at the KEGG level 3 (Figure 3C).

### 3.4. Identifying a Bacterial Signature in PCa Fecal Samples

**Figure 4:**
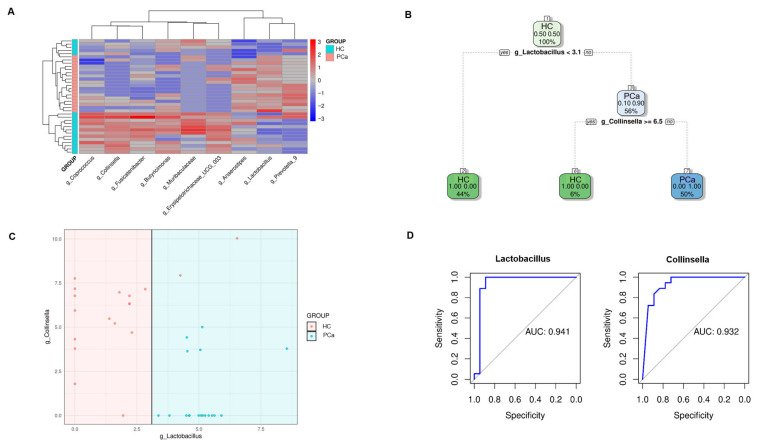
Identification of a bacterial signature in PCa patients. Hierarchical clustering of the genera differentially represented between PCa and HCs according to the Mann–Whitney test and Benjamini–Hochberg correction (**A**). Decision tree structure based on *Collinsella* and *Lactobacillus* relative abundances in PCa and HC (**B**). Plot depicting how fecal samples are clusterized into two classes according to the relative abundances of *Lactobacillus* and *Collinsella* (**C**). AUC analyses assessing the performance of *Lactobacillus* and *Collinsella* models (**D**).

To identify a bacterial signature in fecal samples from PCa patients, the bacterial genera, differentially represented between the two groups, whose hierarchical clustering is reported in Figure 4A, were analyzed using a recursive partitioning tree. As depicted in Figure 4B,C, applying this algorithm revealed that the two genera could accurately distinguish between PCa and HCs. Specifically, a *Lactobacillus* relative abundance < 3.1% corresponded to healthy samples, correctly identifying 16 out of 18 samples. Moreover, samples with both a *Lactobacillus* relative abundance > 3.1% and *Collinsella* relative abundance < 6.5% were classified as belonging to PCa, correctly identifying all 18 tumor cases. The performance of these two bacteria was assessed through AUC analyses. The *Lactobacillus* model demonstrated excellent overall discriminatory power, with an AUC of 0.941 (95% CI: 0.838–1.000). At the optimal threshold of 3.1, the model achieved perfect sensitivity (100%) while maintaining a high specificity (88.9%). Furthermore, *Collinsella* too showed an excellent discriminatory ability with an AUC of 0.932 (95% CI: 0.850–1.000). The optimal threshold of 5.107 yielded perfect sensitivity (100%) and good specificity (72.2%).

### 3.5. Correlation of the Microbial Data with Clinical Pathological Features

**Figure 5:**
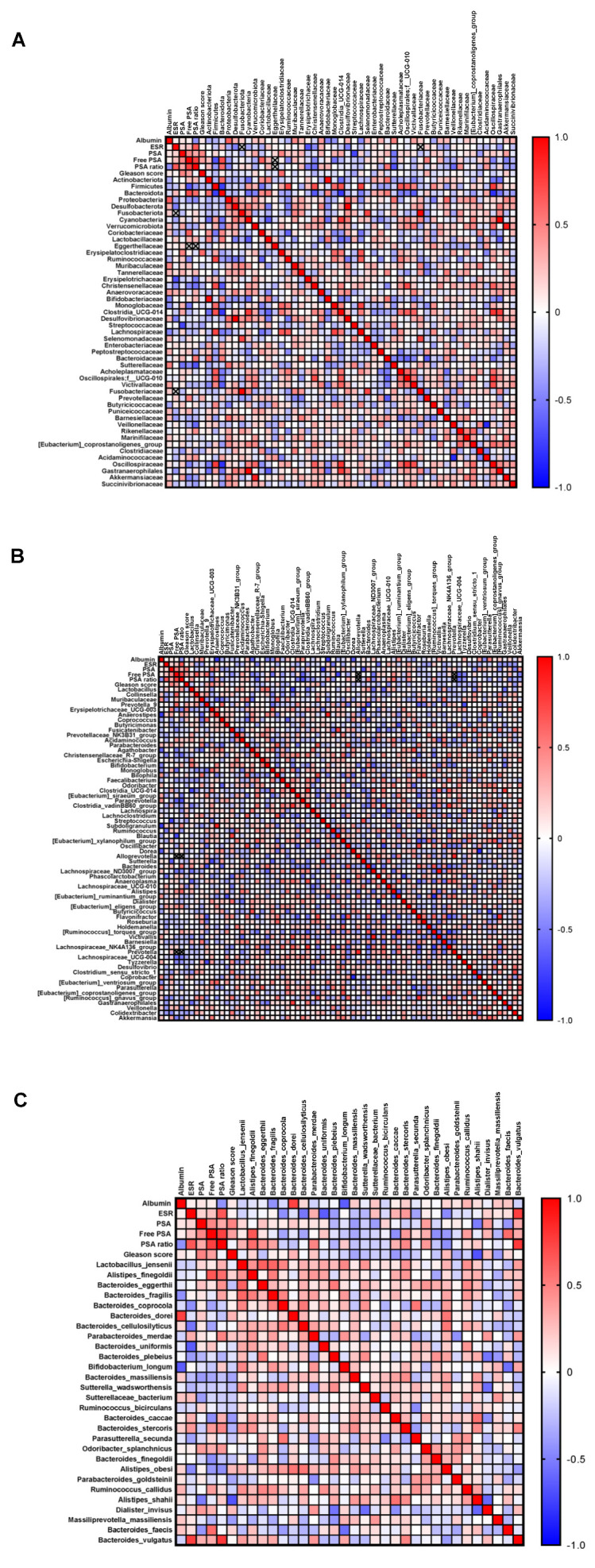
Correlation analysis between clinical parameters and gut microbiota. Spearman’s correlation of the quantities of clinical parameters with intestinal bacteria identified at phylum and family (**A**), genus (**B**), and species (**C**) level. Shades from blue (negative correlation) to red (positive correlation) indicate the value of the R correlation coefficient.

At enrolment, PCa patients underwent routine blood tests. The median results of such exams were within the reference range except for the serum albumin, whose median level was below the normal range, and ESR, PSA, free PSA, and PSA ratio, whose median levels were above the reference interval. In order to investigate whether the gut microbiota could have any impact on the clinical course of the disease, the bacteria identified in the PCa gut microbiota at each taxonomic level were correlated with these patients’ laboratory parameters and with the Gleason score. A number of significant correlations were found at the phylum, family (Figure 5A), genus (Figure 5B), and species level (Figure 5C). Among the strongest ones, PSA levels were negatively associated with *Streptococcus* (r = −0.73) and *Sutterella* (r = −0.64); free PSA was positively correlated with Bacteroidetes (r = 0.70) and *Alistipes finegoldii* (r = 0.66) and negatively correlated with Firmicutes (r = −0.67) and *Blautia* (r = −0.72); and the PSA ratio was directly associated to *Bacteroides vulgatus* (r = 0.77) and indirectly associated to Sutterellaceae (r = −0.67) and *Paraprevotella* (r = −0.66). In addition to prostate specific markers, albumin was positively correlated with Proteobacteria (r = 0.64) and *Bacteroides dorei* (r = 0.74), and negatively correlated with *Anaerostipes* (r = −0.77), *Subdoligranulum* (r = −0.76), *Dorea* (r = −0.63), and *Bifidobacterium longum* (r = −0.64); and the ESR showed an indirect correlation with Erysipelotrichaceae (r = −0.69) and *Bacteroides uniformis* (r = −0.64), and a positive correlation with *Bacteroides vulgatus* (r = −0.75). Finally, the abundance of *Alistipes shahii* was found to negatively correlate with the Gleason score (r = −0.68).

## 4. Discussion

PCa is a common tumor among men but the pathophysiological factors contributing to its onset and progression are still not fully understood. The test of choice for PCa screening is represented by PSA blood measurement, although no clear evidence has been produced so far that the usefulness of this screening tests outweighs its disadvantages, including false positives and over-diagnosis [29,30]. Therefore, the search for new biomarkers or tools which can discriminate PCa patients from healthy subjects and help in understanding the molecular mechanisms involved in the pathogenesis of such disease is still needed. In the current study, we aimed to assess whether the gut microbiota structure and profile of PCa patients could be used as a marker capable of discriminating affected individuals from healthy ones. We found that the alpha-diversity metrics describing both the richness and evenness of the gut bacterial populations was significantly lower in PCa patients as compared to the ones in healthy subjects. This observation hints at a dysbiotic condition in cancer patients since a high gut diversity is generally recognized as an indicator of health, and it is in agreement with the results of a recent meta-analysis asserting a lower diversity in the gut microbiota of PCa carriers with respect to unaffected individuals through different studies [31]. In addition, a significant compositional dissimilarity was observed between PCa and HCs, as assessed by a beta-diversity analysis and by a differential abundance analysis, which revealed a number of microbial taxa as significantly over- or under-represented in cancer carriers. Some of the changes observed in our study are consistent with previous findings, such as the over-representation of Firmicutes and Actinobacteria and under-representation of *Prevotella* in PCa patients compared to men without cancer [31]. Alterations in the gut microbial profile can be involved in PCa pathogenesis through their ability to intervene in metabolic processes. Indeed, in our study, the inference on the functional profiles of the gut microbiota suggested a number of metabolic pathways were differentially represented in the two study groups. Worthy of note is the enrichment of the biosynthesis function of steroid hormones in PCa patients, which has been already described in a study by Kalinen et al. [10]. This finding is of great interest since circulating levels of sex steroid hormones, especially androgens, play an essential role in PCa development and progression [32]: the testosterone can bind directly to the androgen receptor, which is expressed by the majority of prostate cancers or can be converted into the more active metabolite dihydrotestosterone, activating transcriptional pathways which fuel cancer [10,33,34]. Although androgens in men are mainly produced by the testes and the adrenal gland, the microbiota has also been shown to regulate their metabolism [35], thus supporting a role for the gut bacteria in sustaining PCa. Another intriguing function predicted to be differentially regulated between PCa patients and HCs in our study is represented by the Krebs cycle, which we found to be activated in cancer-bearing men. Unlike the majority of cells that consume citrate in the Krebs cycle to fuel energy production through oxidative phosphorylation, normal prostate cells tend to save citrate for secreting it as a component of semen. In malignant prostate cells, however, this phenotype is reversed and citrate is used in the Krebs cycle to produce energy [36]. For this reason, the up-regulation of the Krebs cycle in samples from PCa patients compared to HCs could suggest a contribution of the gut microbiota to sustaining this metabolic pathway. Future studies performing a metagenomics and/or metabolomics analysis would be desirable in order to support the current functional speculations.

In order to investigate whether certain gut bacteria could be used as biomarkers for PCa detection or prognosis, a two-factor decision tree analysis was performed on the differentially represented microbial genera and the whole gut microbial profiles of patients were correlated to parameters generally assessed in PCa clinical management. Interestingly, the decision tree algorithm allowed us to accurately discriminate between PCa patients and healthy individuals based on the relative abundances of *Lactobacillus* (increased in PCa) and *Collinsella* (decreased in PCa). The exact role of these bacteria in the pathogenesis of PCa still needs to be investigated. Lactobacilli are generally regarded as beneficial microbes; nevertheless, they have been suggested to promote certain types of malignancies, including gastric, breast, and pancreatic cancers, by inhibiting the host immune system [37,38,39]. As for PCa, the *Lactobacillus* genus was previously described to be enriched in the prostatic tissues of patients without a biochemical recurrence as compared to the ones with a biochemical recurrence, but no information is available about its abundance in the gut, and nothing is known about the origin of the prostatic microbiota [40].

In addition, a certain number of bacterial taxa was found to positively or negatively correlate with patients’ clinical parameters. Among all, a strong negative correlation between the species *Alistipes shaii* and the Gleason score emerged. This result could seem conflicting with a previous paper by Matsushita et al., in which the genus *Alistipes* was found to be significantly increased in Japanese PCa patients with a high Gleason score [41]. A plausible explanation is that gut microbiota composition and its association with PCa may differ according to ethnicity [42]. In addition, our indirect correlation with the Gleason score only concerned the *A. shaii* species, and not the entire *Alistipes* genus. Moreover, *A. shaii* is a short-chain fatty acid producer expected to play a beneficial anti-inflammatory role in health, as confirmed in a mouse model of colitis [43], and by its decreased abundance in patients suffering from intestinal bowel diseases [44].

The current study presents some limitations: the size of the study population is small and metagenomics or metabolomics analyses providing biochemical mechanisms underlying the microbial differences observed are missing. Further prospective studies and clinical validation supporting the current findings are needed. Nevertheless, the findings of our study suggest a future potential for the gut microbiota to be exploited as a novel biomarker and could guide research towards further investigations aimed at developing new non-invasive microbiota-based supportive tools in the screening, diagnostic, and prognostic framing of PCa patients.

## Figures and Tables

**Table 1:** Anthropometric and lifestyle characteristics of the study population.

Variable	Category	All Subjects (N = 36)	Healthy Controls (N = 18)	PCa Patients (N = 18)	*p*-Value
Anthropometric characteristics	-	-	-	-	-
Height (cm)	Median [IQR]	172.0 [167.0–178.0]	172.0 [168.5–177.5]	171.0 [166.3–177.5]	0.782 ^
Body weight (Kg)	Median [IQR]	79.0 [72.0–87.0]	85.0 [74.5-90.0]	75.0 [72.0–85.0]	0.123 ^
BMI (Kg/m^2^)	Median [IQR]	26.8 [24.6–28.7]	28.1 [25.7–29.2]	25.7 [24.6–27.6]	0.117 ^
Age (years)	Mean ± SD	65.6 ± 6.5	62.1 ± 4.4	69.1 ± 6.5	0.001 *
Lifestyle info	-	-	-	-	-
Diet—N (%)	Mediterranean	29 (80.6)	13 (72.2)	16 (88.9)	0.098 ^§^
	Free/hyperglucidic/hyperproteic	3 (8.3)	1 (5.6)	2 (11.1)	
	Not known	4 (11.1)	4 (22.2)	0 (0.0)	
Smoking status—N (%)	No	23 (63.9)	9 (50.0)	14 (77.8)	0.074 ^§^
	Yes	9 (25.0)	5 (27.8)	4 (22.2)	
	Not known	4 (11.1)	4 (22.2)	0 (0.0)	
Alcohol consumption—N (%)	No	21 (58.3)	10 (55.6)	11 (61.1)	0.088 ^§^
	Yes	11 (30.6)	4 (22.2)	7 (38.9)	
	Not known	4 (11.1)	4 (22.2)	0 (0.0)	

* *p*-value from the two-sample test; ^ *p*-value from Mann–Whitney U test; ^§^ *p*-value from Chi-Square test with Yates’ continuity correction. IQR: Interquartile Range.

## Data Availability

The data presented in this study are openly available in ArrayExpress [https://www.ebi.ac.uk/fg/annotare/edit/20091/] under the accession code E-MTAB-14719.

## Abbreviations

PCa	prostate cancer
PSA	prostate-specific antigen
HCs	healthy controls
ESR	erythrocyte sedimentation rate
BMI	body mass index
FDR	false discovery rate
